# Intensifier of urban economic resilience: Specialized or diversified agglomeration?

**DOI:** 10.1371/journal.pone.0260214

**Published:** 2021-11-29

**Authors:** Mingdou Zhang, Qingbang Wu, Weilu Li, Dongqi Sun, Fei Huang

**Affiliations:** 1 School of Regional and Urban Sciences, Shanghai University of Finance and Economics, Shanghai, China; 2 School of Economics, Dongbei University of Finance and Economics, Dalian, China; 3 Institute of Economics and Social Development, Dongbei University of Finance and Economics, Dalian, China; 4 Institute of Geographic Sciences and Natural Resources Research, Chinese Academy of Sciences, Beijing, China; 5 School of Marxism, Shanghai University of Finance and Economics, Shanghai, China; China University of Mining and Technology, CHINA

## Abstract

With increased uncertainty and instability worldwide, how to enhance the urban economy resilience effectively has become one main issue for urban economic development. Based on the measurement of the economic resilience of 241 cities at the prefecture level and above in China using the sensitive index method, we scrutinize the impact of industrial specialization agglomeration and diversification agglomeration on urban economic resilience. Results indicate that, during the impact resistance period, industrial diversification agglomeration, especially related industrial diversification agglomeration, can enhance urban economic resilience, whereas industrial specialization agglomeration has no positive effect. In contrast, during the period of recovery and adjustment, industrial specialization agglomeration can improve urban economic resilience, and industrial diversification agglomeration, especially related industrial diversification agglomeration, has no positive effect. Further analysis indicates that, under the interaction of specialization and diversification agglomerations, the effect of industrial agglomeration on urban economic resilience depends on the type of dual industrial agglomeration, showing remarkable heterogeneity. This study may provide useful references for policy makers concerned with urban resilience.

## 1 Introduction

In recent years, the global economy has been slowing down and facing upgraded risks. Uncertainty and instability are increasing, and traditional and non-traditional security issues are growing [1]. According to the report of the "world economic situation and prospects", we can find that the global average economic growth rate in the past ten years is only 2.5%, of which the global economic growth rates in 2016 and 2019 were only 2.2% and 2.3%. Affected by the coronavirus pandemic in 2020, the global economy has shrunk by 4.4%, which is more than 2.5 times that of the 2008 global financial crisis [2]. The Asian financial crisis in 1997, the international financial crisis in 2008, the SARS in 2003, and the coronavirus pandemic in 2020 have brought far-reaching impacts on urban economic development. When faced with a crisis, the economies of some cities become vulnerable and suffer severely, whereas the economy of some other cities shows strong resilience and even take the crisis as an opportunity for industry upgradation. Thus, what are the reasons behind the dramatic difference in performance? The answer to this question is not only of theoretical interest but also of great practical significance for urban economic development. Under the background of urban agglomeration worldwide, this study explores the intensification of urban economic resilience from the perspective of industrial agglomeration to determine the role of different types of industrial agglomeration in urban economic crisis.

The capability of the urban economy to resist external shocks and recover is referred to as *urban economic resilience*, which was introduced in the field of spatial economics by Fujita and Thisse, Reggiani et al. [3,4]. It gradually becomes an important concept in the field of economic geography. From the perspective of regional economics, Edward defines urban economic resilience as the urban economy that maintains or restores to its original equilibrium state after undergoing external impacts, that is, the urban economy is not affected or can be recovered rapidly from the impact [5]. Martin et al. define urban economic resilience from four dimensions, namely, the capability to resist and absorb shocks, the extent of the response to shocks, the capability of the urban economic system to make changes in the integration of internal resources and structural adjustment after the impact, and the return to the original growth path or creation a new growth path, which has been well recognized by academics because of its comprehensiveness [6]. It can be seen that economic resilience is an adjustment ability involving multiple stages, that is, not only refers to the resistance and recovery to shocks; but emphasizes the adaptive adjustment formed by the rearrangement of production factors after the crisis [7]. In other words, when the city’s economy is hit, it will respond to hit and follow the development trend, and even break the locked state formed by the medium and long-term solidification, thereby adjusting the long-term balanced development path. This paper also adopts this concept and believes that when the urban economic system responds to external shocks, it will redistribute resources in a more effective way, adjust the industrial structure, and form an adaptive new growth path.

The literature on urban economic resilience can be divided into two main stages [8]. The first stage (2002–2010) mainly refers to the buildup of concepts and fundamental theories of economic resilience from other research fields. The second stage (2010–present) is marked by empirical research on economic resilience based on quantitative analysis methods. The global financial crisis in 2008 has drawn more academics’ attention to urban economic resilience, which leads to more comprehensive studies. Existing literature generally finds industrial structure as an important influencing factor for urban economic resilience. Davies, Xu and Warner [9,10] examine the economic resilience of Europe and the United States, respectively, from the perspective of industrial specialization agglomeration. The results indicate that regions with higher manufacturing specialization agglomeration possess worse urban economic resilience. Based on the empirical analysis of Munich, the metropolitan area of the U.S., Ohio in the U.S., and other regions, Evans and Karecha, Doran and Fingleton, and Brown and Greenbaumfind that the level of industrial diversification has a positive effect on the economic resilience of the city under external impact [11–13]. Castaldi et al.further analyze the effect of industrial diversification on the regional economy by decomposing industrial diversification into related and unrelated components [14]. Studies on urban economic resilience from other aspects include Fingleton et al., Brakman et al., and Todo et al. [15–17].

Han et al. studied the impact of industrial relevance on urban economic resilience based on the concept of network, and believed that improving the central position of the local industrial structure in the urban economy is beneficial to resist future risk shocks [18]. However, it did not thoroughly analyze the impact of urban heterogeneity and industrial relevance on urban economic resilience. According to the theory of evolutionary economic geography, the resilience and post-crisis resilience of different economies are heterogeneous [19,20]. Meanwhile, the industrial structure of cities will evolve over time [21,22]. In other words, the differences in industrial structure, size, infrastructure level, and spatial location of different cities make the same impact on the production, employment, and income of different cities. For example, Chelleri et al. [23]used the case analysis of Kampala slums to show that urban economic resilience is heterogeneous in different urban scales; Hundtc and Holtermann studied the differences in specific national systems and regional determinants of urban economic resilience in 249 NUTS-2 regions nested in 22 European countries [24]; Tan et al. used 114 RBCs in China and found that urban economic resilience also shows huge heterogeneity in specific industries [25]. It can be seen from this that when the urban economy suffers an impact, it is not a simple static adjustment, but a dynamic evolution based on its own industrial structure characteristics and industrial agglomeration level. Therefore, when analyzing the effect of industrial agglomeration on urban economic resilience, we must fully consider its heterogeneous characteristics. From the perspective of industrial agglomeration level, this paper thoroughly examines the impact of industrial agglomeration combinations at different industrial agglomeration levels on urban economic resilience.

Despite the important role played by China’s economy in the world, the literature on urban economic resilience of China remains limited, and few empirical studies involve urban economic resilience, and the research objects are mainly concentrated in individual provinces and regions [26–28]. Considering the unique attributes and policy environment of China’s urban economic development, the applicability of existing conclusions based on data from developed economies are yet to be examined. The existing literature generally focuses on one aspect of industrial agglomeration, specialization agglomeration or diversification agglomeration, and few studies have explored the interaction between the two types of agglomeration and the resulting heterogeneity.

Given the above, the present study proposes a unified analytical framework to scrutinize the effects of industrial specialization agglomeration and industrial diversification agglomeration on urban economic resilience simultaneously and contributes to the existing literature from the following aspects. First, based on the practice of urban economic development in China, this study conducts a quantitative comparative study on the effect of industrial specialization agglomeration and diversified agglomeration on urban economic resilience. Moreover, the industrial diversification agglomeration is decomposed into related and unrelated diversification agglomerations to explore the differences in relationships between the two types of diversified agglomerations and urban economic resilience. Second, considering the differences in the level of industrial agglomeration between cities, this study investigates the heterogeneity in the effect of industrial specialization and diversification agglomeration on urban economic resilience and analyzes the impact of the agglomeration level. Third, based on the perspective of dual industrial agglomeration types, the samples are divided into four quadrants of "high-high, high-low, low-high, and low-low" with two industrial agglomeration levels. This paper further analyzes the utility heterogeneity of the two types of industrial agglomeration on urban economic resilience, in order to analyze the interaction between specialized agglomeration and diversified agglomeration.

The rest of the paper is organized as follows. Section 2 provides the theoretical mechanism analysis. Section 3 describes the measurement and stage division of urban economic resilience. Section 4 presents the research design. Section 5 shows the analysis of empirical results. Section 6 concludes the paper.

## 2 Theoretical mechanism analysis

The industrial structure is the foundation of the urban economy and thus is an important influencing factor of urban economic resilience. As the two types of industrial agglomeration, specialized and diversified agglomerations can have different effects on urban economic resilience. The core of industrial specialization agglomeration is the Marshall externality, which emphasizes that the agglomeration of the same industry in a specific region can promote information exchange and technology diffusion and share the scale effect of the specialized labor force and intermediate input, to improve rapidly the productivity and expand the production scale of the leading industry [29]. Industrial specialization agglomeration is essentially the result of the internal economic interaction of specific industries in cities, and its effect on urban economic resilience mainly consists of two aspects. On the one hand, the core characteristics of industrial specialization agglomeration are highly specialized infrastructures and closely related industrial chains, which also mean high sunk costs and industrial barriers that are not easy to exit. Consequently, industrial specialization agglomeration can easily lead to cognitive locking, which means the urban industry cannot break through the industrial and spatial boundaries and develop into a closed system, which will restrain the innovation and flexibility of the city. Therefore, when the urban economy undergoes a crisis, the highly related industrial structure will produce a chain reaction and thus exacerbate the impact on the urban economy. On the other hand, industrial specialization agglomeration can effectively utilize the knowledge spillover effect and the sharing effect of information and equipment in the industry and develop the local economy efficiently to reduce the marginal and congestion costs of urban industrial development, thus forming the scale economy effect. Therefore, after a crisis, industrial specialization agglomeration can recover the production efficiency in a short time and thus rapidly restore the vitality of the urban economy.

The core of industrial diversification agglomeration is the Jacobs externality, which emphasizes the spillover effect of information, knowledge, and technology between different industries, as well as collaborative innovation and all-round development across industries [30]. The industrial diversification agglomeration is essentially the result of the cross-sector economic interaction between urban industries, and its influence on urban economy resilience mainly consists of two aspects. On the one hand, the diversified industrial structure can realize the diversification of urban economic risks [31,32] over different types of industries with different flexible demand, export orientation, labor and capital intensity, and external competitive risk, effectively reducing the impact of different types of shocks. Meanwhile, by promoting the formation and diffusion of new knowledge and technologies, diversified agglomeration can provide more powerful technology in support of urban economy development, which can make urban industries more competitive and accelerate the incubation, derivation, and growth of emerging industries [33] and provide necessary technical preparation for the transformation and adjustment of industrial structure in case of crisis. Moreover, industrial diversification agglomeration can improve the matching efficiency of human resources, reduce the outflow of the labor force, and improve the stability of the human resources market. Therefore, industrial diversification agglomeration can enhance the resistance to external shocks for the urban economy and stabilize the internal environment of the urban economy in crisis. On the other hand, industrial diversification agglomeration can be taken as a kind of diversified investment. Although it can provide an efficient and multi-channel development path for cities, it also requires high cost bearing capability of the urban economy. Diversified agglomeration will not only weaken the localization economy produced by specialized agglomeration but also increase the congestion cost of the urban industry. Once the suitable production process cannot be found, the urban economy is prone to stagflation. Therefore, after the crisis, industrial diversification agglomeration will aggravate the scarcity of urban resources and thus hinder the recovery and adjustment of the urban economy.

Industrial diversification agglomeration can be further decomposed into related and unrelated industrial diversification agglomerations [34]. The basic idea behind is that related diversification agglomeration is beneficial to enhance the spillover effect of knowledge and technology, whereas unrelated diversification agglomeration can produce the portfolio effect [34,35]. The two kinds of industrial diversification agglomeration have different effects on urban economic resilience. On the one hand, the correlation between industries provides favorable conditions for the spillover of knowledge and technology, which can effectively reduce the cost of industrial innovation and improve the overall level of innovation. Diversified technology integration and cross-innovation are the main ways to enhance the resilience of the urban economy [14,36]. In contrast, producing a high level of spillover effect between unrelated industries is difficult, and the industrial collaborative innovation becomes more difficult. On the other hand, when the urban economy fluctuates, the closely related industrial structure can easily trigger chain reactions, that is, the impact will spread rapidly from one industry to other related industries, further exacerbating the urban economic fluctuations and possibly causing systemic risks. In contrast, unrelated industries have different flexible demands and life cycles, and no close input-output relationship exists between industries. Therefore, unrelated industrial diversification agglomeration can bring the portfolio effect to the urban economy and effectively diversify risk.

From the above analysis, it can be found that the impact of industrial specialization and diversification agglomeration on urban economic resilience is not a pure one-way effect, nor can it be analyzed with the idea of classical dichotomy. When the urban economy suffers from external shocks, industrial specialized agglomeration can maximize economies of scale and local economic benefits through Marshall externalities, thereby helping the urban economy to get rid of the crisis. However, industrial specialization agglomeration, along with the rise and fall of specific industries and technologies, will inevitably lead the urban economy into the next round of risks that cannot withstand external shocks. In order to avoid the huge risks brought by industrial specialization and agglomeration to the urban economy, all cities will choose to develop diversified agglomeration, and then use the “portfolio effect” and “innovation incentive effect” brought by diversified agglomeration to resist the next round of risks Shock. However, the resources of each city are limited, which means that the development of diversified cities will inevitably weaken or abandon the important benefits of professional agglomeration to urban economic recovery. From this, we can find that cities need to weigh the pros and cons of industrial specialization and diversified agglomeration for urban economic development both in the impact resistance period and in the recovery period. The result of this choice will lead to the development direction of the city’s economy to a certain extent, and even determine the potential of the city’s future development to a certain extent.

## 3 Measurement and stage division of urban economic resilience

The two methods for measuring urban economic resilience are index system and sensitivity index. The indicator system method was first proposed by Briuglio et al. [37] and has been under development [38,39]. However, the index system method has two inborn deficiencies. One is that the selection of index variables and weight functions has no unified criteria, in addition to the arbitrary weighting of variables–is that they do not typically consider the interaction or tradeoffs between variables. The other is that most indicators selected explain the reasons for the urban economic resilience rather than its measurement, easily leading to causal confusion. In recent years, the sensitivity index method proposed by Martin et al. has been widely used in studies of urban economic resilience because of its objectivity [40]. The sensitivity index method divides urban economic resilience into two periods, namely, the recession period defined by the national economy from peak to trough and the recovery period defined by the national economy from trough to peak, which is in line with the research needs of the present study.

After cities undergo external shocks, the biggest adjustment is the urban labor force. In other words, cyclical fluctuations in employment are often more predictive than cyclical fluctuations in output [40]. As a core variable of the urban economy, employment is closely related to the initial formation of cities, the deduction of basic sector models, and the formulation of urban development policies, and the level of employment is sensitive to the impact of the external environment. The labor market is also closely related to the industrial structure. Lazzeretti et al., Firgo and Mayerhofer conducted research in Italy and Austria, respectively, and found that there is a high correlation between urban employment levels and industrial development [41,42]. Although GDP growth and investment in fixed assets can also reflect the urban economic volatility after the shock, they can be greatly influenced by national policy and institutional mechanisms and cannot accurately reflect the urban economic resilience and differentiate between the stages of impact resistance and recovery adjustment. Taking the above into consideration, this study adopts the sensitive index method to measure the urban economic resilience based on the urban employment level. Considering the error accumulation effect of multi-period prediction, the index measure is built on a single-period prediction. Under the premise that the whole national economy is under the impact of a crisis, based on the annual employment growth rate of city *r* in year *t* and the national average employment change rate in the following year, the counterfactual employment change rate in the following year can be constructed as follows:

$${\left(\Delta \omega_{r,t+1}\right)}^{C}=\sum_{i=1}^{N}\upsilon_{t+1}\omega_{rt}^{i},$$ (1)

where *υ*_*t*+1_ represents the annual national average employment change rate in year *t* and $\omega_{rt}^{i}$ represents the annual employment growth rate of industry *i* (*i =* 1,…, N) in city *r* in year *t*. The economic resilience of city *r* in year *t* can then be defined as follows:

$$\xi_{rt}=\frac{{\left(\Delta \omega_{r,t+1}\right)}^{A}-{\left(\Delta \omega_{r,t+1}\right)}^{C}}{\left|{\left(\Delta \omega_{r,t+1}\right)}^{C}\right|},$$ (2)

where (Δ*ω*_*r*,*t*+1_)^*A*^ represents the actual employment change rate of city *r* in year *t* and *ξ*_*rt*_ reflects the resilience of city *r*’s economy relative to the averaged national economy under the condition that the whole economy of the country suffers from impacts. A positive *ξ*_*rt*_ indicates that the city’s economic resilience to shocks and recovery adjustment is higher than the national average, and vice versa.

Different types of industrial agglomeration have different effects on urban economic resilience at different stages when urban economy undergoes impacts. Thus, this study examines the shock resistance period and the recovery adjustment period separately. Regarding the demarcation between the two stages, the existing literature defines the year with negative sample mean of urban economic resilience as the shock resistance period and the year with positive sample mean of urban economic resilience as the recovery and adjustment period [28]. The choice of demarcation should also account for the actual situation of China’s economic development. Since the third quarter of 2008, the global financial crisis has begun to increasingly impact some domestic cities and industries. Considering the time lagging effect of the shock transmission mechanism and the specific performance of China’s labor and employment market, concluding that the financial crisis has caused a substantial impact on China’s urban economy since 2009 is reasonable. The Chinese government implements the “four trillion” plan, which effectively restrains the further spreading of the impact of the global financial crisis. As a result, China’s economic growth rate rebounded briefly in 2010, when the mean of urban economic resilience also changed from negative to positive. In 2014, General Secretary Xi Jinping formally proposed the definition of “new normal economy” at the Central Economic Work Conference, marking that China’s economy officially entered a new stage in 2015. The mean of urban economic resilience measured in 2015 is negative. Therefore, this study considers 2015 and later years as a new economic cycle. To sum up, 2009 is taken as the shock resistance period, and 2010–2014 is taken as the recovery and adjustment period. In 2009, the mean value of the economic resilience of 241 cities at and above the prefecture level was −0.042, whereas the mean values of the economic resilience of cities from 2010, 2011, 2012, 2013, and 2014 are 3.5412, 0.0403, 4.0704, 1.1308, and 1.0752, respectively.

## 4 Methods

### 4.1 Dependent variable

#### 4.1.1 Explained variable

The explained variable is urban economic resilience, including the shock resistance period and the adjustment recovery period. The specific calculation method is detailed in Section 3.

#### 4.1.2 Explanatory variables

The explanatory variables are industrial specialization and diversified industrial agglomeration.

(1) Industrial specialization agglomeration. The level of industrial specialization agglomeration can be measured by calculating the city specialization index [33]. Considering that the specialization of different cities may belong to different industry sectors and thus may be incomparable, the present study constructs the specialization index based on the location entropy of the industry with the most employment in the city as follows:

$$SP_{rt}=\max(\frac{\delta_{rt}^{i}}{\gamma_{t}^{i}}),$$ (3)

where $\delta_{rt}^{i}$ is the employment proportion of industry *i* in city *r* in year *t*, $\gamma_{t}^{i}$ is the national employment proportion of industry *i* in year *t*, and the proportion of the two is the location entropy. The larger the *SP*_*rt*_, the higher the industrial specialization level of the city *r* in year *t*.

(2) Diversified industrial agglomeration. The level of diversified industrial agglomeration has a few measures, such as the Gini coefficient, EG index, and the inverse of the Hirshman-Herfindahl index, each of which has its advantages and disadvantages. To measure the level of the diversified agglomeration of different types of industries with consistency, this study uses the entropy index as the measure of the level of diversified industrial agglomeration [34] as follows:

$$DIV_{rt}=\sum_{i=1}^{N}\theta_{rt}^{i}\ln(\frac{1}{\theta_{rt}^{i}}),$$ (4)

where $\theta_{rt}^{i}$ is the employment proportion of industry *i* in city *r* in year *t*. The larger the *DIV*_*rt*_, the higher the diversified industrial agglomeration level.

To analyze further the effect of the related and unrelated diversification agglomeration on urban economic resilience, this study decomposes the entropy index of the diversified industrial agglomeration into two components. Suppose all sectors have g sub-sectors, where *g* (*g* = 1, 2,…, G), the diversified industrial agglomeration entropy index can be decomposed as follows:

$$\begin{array}{l} DIV_{rt}=\sum_{i=1}^{N}\theta_{rt}^{i}\ln(\frac{1}{\theta_{rt}^{i}})=\sum_{g=1}^{G}\sum_{i\in g}\theta_{rt}^{i}\ln(\frac{1}{\theta_{rt}^{i}})\\ \;=\sum_{g=1}^{G}\sum_{i\in g}\theta_{rt}^{i}[\ln(\frac{\theta_{rt}^{g}}{\theta_{rt}^{i}})+\ln(\frac{1}{\theta_{rt}^{g}})]\\ \;=\sum_{g=1}^{G}\left[\sum_{i\in g}\theta_{rt}^{g}(\frac{\theta_{rt}^{i}}{\theta_{rt}^{g}})\ln(\frac{\theta_{rt}^{g}}{\theta_{rt}^{i}})\right]+\sum_{g=1}^{G}\left[\sum_{i\in g}\theta_{rt}^{i}\ln(\frac{1}{\theta_{rt}^{g}})\right]\\ \;=\sum_{g=1}^{G}\theta_{rt}^{g}[\sum_{i\in g}\left(\frac{\theta_{rt}^{i}}{\theta_{rt}^{g}})\ln(\frac{\theta_{rt}^{g}}{\theta_{rt}^{i}}\right)]+\sum_{g=1}^{G}\theta_{rt}^{g}\ln(\frac{1}{\theta_{rt}^{g}})\\ \;=RV_{rt}+UV_{rt}, \end{array}$$ (5)

where *RV*_*rt*_ is the level of related variety, measuring the degree of diversification of sub-industries within the major sectors of the city *r* in year *t* and *UV*_*rt*_ is the level of unrelated variety, measuring the degree of diversification of the industrial sectors with weak linkages to the major sectors of city *r* in year *t*.

#### 4.1.3 Control variables

Following the literature [28,43–45], this study selects a sequence of control variables as follows: (1) Degree of government intervention (*Gover)*. The imperfect market mechanism and government-led advancement are important characteristics of China’s urbanization process. Therefore, the degree of government intervention influences the development of the urban economy. Moreover, the government can also affect urban economic resilience through institutional policies [46]. This study selects the proportion of government budget expenditure in GDP as the measure of the degree of government intervention. (2) Urban economic development level (*Eco*). The level of urban economic development directly affects the capability of the urban economy to resist shocks and adjust and recover. This study uses GDP per capita to measure the level of urban economic development. (3) The level of openness (*Fdi*). The level of urban openness has a relatively complicated relationship with urban economic resilience. The higher the level of openness, the higher the city is related to the outside world and subject to more external shocks. Meanwhile, cities with higher levels of openness generally have higher degrees of urbanization and are more resistant to crises. We take the proportion of foreign direct investment in GDP used in cities as the measure of the level of openness. (4) Market size. New economic geography believes that the impact of the urban economy is directly mapped on the market scale. The market size is difficult to be measured by a single proxy. This study uses population density (*Popden*) and the total retail sales of consumer goods (*Market*) to measure the market size. (5) The level of urban human capital (*Humcap*). High-quality human capital is an important factor in enhancing urban economy resistance [47]. Innovation and adaptability are important foundations for accelerating adjustment recovery and industrial upgrading. This paper takes the number of college students per 10,000 students as the measure. (6) Urban infrastructure level (*Rd*). Infrastructure is a necessary foundation for urban economic resilience and a constraint on the speed of urban economic adjustment recovery. Per capita urban road area is used as the measure. (7) Urban innovation level (*R&D*). Innovation is an important force for urban economic development and is also the core foundation for dynamic adjustment and upgrading after the impact. The number of invention patent applications per 10,000 people is taken as the measure. (8) Fixed asset investment level (*Fix-cap*). Modern cycle theory believes that investment fluctuations are the main cause of economic fluctuations. Investment demand management, as an important tool of government macro-control, also has an important impact on the stable development of the urban economy. Therefore, we use the proportion of the fixed capital investment of the whole society in the city’s GDP as the measure.

### 4.2 Model construction

Given that the impact resistance period is only one year, the cross-sectional regression is used for analysis. Regarding the adjustment recovery period, this study uses the dual-effect panel regression model to analyze the impact of different types of industrial agglomeration on urban economic resilience as follows:

$$\xi_{rt}=\beta_{0}+\beta_{1}SP_{rt}+\beta_{2}X_{rt}+u_{r}+v_{t}+\varepsilon_{rt}$$ (6)

$$\xi_{rt}=\beta_{0}+\beta_{1}DIV_{rt}+\beta_{2}X_{rt}+u_{r}+v_{t}+\varepsilon_{rt}$$ (7)

where *X*_*rt*_ represents the control variables that may affect the region economic resilience; *u*_*r*_ and *v*_*t*_ are the fixed effect of individual and time, respectively; and *ε*_*rt*_ is the error term. To examine further the effect of different types of diversified industrial agglomeration on urban economic resilience, in the subsequent empirical analysis, the industrial diversification agglomeration in Formula (7) is replaced by related and unrelated diversification agglomeration.

### 4.3 Date description

This study takes prefecture-level and above cities in China as the research sample. The data are mainly collected from the 2010–2015 *China Statistical Yearbook*, *China City Statistical Yearbook*, and *China Regional Economic Statistical Yearbook*. Statistical bulletins and official websites of relevant government departments are used to search for missing data. The data is further explained as follows: (1) Computing related and unrelated diversification agglomeration requires a clear industry classification criterion. The 19 industry categories specified in the 2003 *China City Statistical Yearbook* is used throughout the study. (2) Following the existing literature [48], the 19 industry categories emerged into six major industries as follows: agriculture, forestry, animal husbandry, and fishery form the primary industries; extractive industries, manufacturing, electricity, gas and water production and supply, and construction form the secondary industries. The financial industry, real estate industry, and leasing and business service industries form the productive service industries; the accommodation and catering industry, resident service and other service industries; and culture, sports, and entertainment form the consumer service industries; transportation, warehousing, post and telecommunications, information transmission, computer services and software, and wholesale and retail trade form the liquid service industries; scientific research, technical service and geological survey industry, water conservancy environment and public facility management industry, education industry, health and social insurance and social welfare industry, public management, and social organization form the social service industries. (3) Subject to the availability and completeness of the data, this study removes Lhasa, Haidong, Hong Kong, Macao, and Taiwan from the sample, resulting in 241 sample cities in total. The descriptive statistics of related variables are shown in Table 1.

**Table 1:** Descriptive statistics of related variables.

Variables	Variables Name	Variable Description	Mean	sd	Min	Max
*RRC*_*1*_	Urban Economic Resilience	Impact resistance period	-0.042	1.839	-7.248	7.025
*RRC*_*2*_		Recovery adjustment period	1.972	3.387	-19.857	18.868
*SP*	Industry specialization agglomeration	Industrial specialization agglomeration level	2.724	3.883	0.098	38.390
*DIV*	Industrial diversified agglomeration	Industrial diversified agglomeration level	2.230	0.227	1.206	2.706
*RV*	related DIV	Level of related DIV	1.017	0.146	0.462	1.357
*UV*	unrelated DIV	Level of unrelated DIV	1.213	0.145	0.609	1.742
*Gover*	Degree of government intervention	Government budget expenditure/urban GDP	0.171	0.077	0.043	0.688
*Eco*	Urban economic development level	Per capita GDP log value	10.400	0.632	4.595	13.060
*Fdi*	The level of opening	The city actually uses foreign direct investment/urban GDP	0.019	0.018	0.001	0.129
*Popden*	Market size	Population density log value	5.773	0.895	1.603	7.727
*Market*		Total retail sales of consumer goods log value	15.200	0.979	12.160	18.380
*HumCap*	The level of urban human capital	The number of college students per 10,000 students log value	4.519	1.170	-0.552	7.147
*Rd*	Urban infrastructure level	Urban road area per capita log value	2.268	0.578	-0.528	4.686
*R&D*	Urban innovation level	Invention patent applications per 10,000 people log value	0.880	2.193	-5.552	11.680
*Fix_cap*	Fixed asset investment level	Social fixed capital investment/urban GDP	0.720	0.221	0.087	1.852

Table 1 shows that the average level of industrial specialization agglomeration and diversified industrial agglomeration in the sample cities are relatively similar. Thus, the regression coefficients are relatively comparable. The standard deviation of industrial diversified agglomeration is much smaller *than* its average, and the gap between the maximum and minimum is not large, indicating that the gap in the level of industrial diversified agglomeration among sample cities is relatively small. In contrast, the standard deviation of industrial specialization agglomeration is greater than its mean, and a huge gap is observed between the maximum and minimum values, indicating that the differences in the level of industrial specialization agglomeration among sample cities are significant. Thus, the current situation of China’s urban economy is accurately reflected.

## 5 Results and discussion

### 5.1 The effects of different types of industrial agglomeration on urban economic resilience

Based on the result of the Hausman test, the fixed-effect model is selected to analyze the urban economic resilience during the period of recovery and adjustment. The regression results are shown in Table 2. The first and second columns of Table 2 show that, after the outbreak of the financial crisis in 2008, the diversified industrial agglomeration has a significant positive effect during the period of impact resistance, whereas specialization agglomeration shows a significant negative influence. Therefore, diversified industrial agglomeration can improve urban economy’s resilience to impacts, but specialization agglomeration will worsen the situation. The mechanism analysis above shows that diversified industrial structure can effectively help urban economy diversify risks, whereas the potential risk contagion chain of specialized industrial structure will continuously intensify the impact on the urban economy.

**Table 2:** The effects of two types of industrial agglomeration on urban economic resilience.

	The period of impact resistance		The period of recovery and adjustment	
	(1)	(2)	(3)	(4)
*SP*	-0.106** (-2.24)		0.183*** (2.69)	
*DIV*		1.374** (2.36)		-4.969*** (-4.34)
*Gover*	4.393* (1.67)	4.405* (1.67)	-3.986 (-0.79)	-3.728 (-0.75)
*Eco*	0.590* (1.71)	0.587* (1.71)	0.316 (0.33)	-0.121 (-0.13)
*Fdi*	-2.002 (-0.30)	1.916 (0.28)	32.180** (2.31)	38.700*** (2.77)
*Fix_cap*	-0.177 (-0.30)	-0.244 (-0.41)	-1.102 (-1.24)	-0.935 (-1.06)
*R&D*	0.143** (2.50)	0.158*** (2.74)	0.576** (2.03)	0.595** (2.10)
*Humcap*	-0.117 (-0.87)	-0.157 (-1.15)	-0.010 (-0.02)	0.067 (0.13)
*Rd*	0.176 (0.70)	0.204 (0.80)	-0.083 (-0.18)	0.033 (0.07)
*Popden*	0.353** (2.01)	0.479*** (2.69)	-1.566 (-0.43)	-0.563 (-0.16)
*Market*	-0.350* (-1.79)	-0.309 (-1.63)	-0.010 (-0.02)	0.189 (0.28)
*_cons*	-2.975 (-0.69)	-7.526* (-1.68)	6.028 (0.27)	13.780 (0.62)
*N*	241	241	1205	1205
*R* ^*2*^	0.048	0.050	0.333	0.341

*Notes*: *t* statistics are shown in parentheses.

*, **, and ***indicate the significance of p-values at 10%, 5% and 1% level, respectively.

The third and fourth columns of Table 2 reveal that, during the period of recovery and adjustment, industrial diversified agglomeration shows a significant negative effect, whereas specialized agglomeration presents a *significant* positive effect. Therefore, specialized industrial agglomeration can effectively help in urban economic recovery and adjustment, whereas diversified agglomeration has exactly the opposite effect. The diversified industrial structure can be viewed as a diversified investment [33]; the production cost required can become unbearable for the urban economy during the recovery and adjustment period. In contrast, the specialized industrial structure can utilize the spillover effect of professional technology to form scale economies in the short term to boost urban economic adjustment and structural transformation. Doran and Fingleton [49] draw a similar conclusion in their study on the economic resilience of the US metropolitan area.

Related and unrelated industrial diversified agglomerations can have different effects on urban economic resilience. Based on the Hausman test results, fixed effect regressions are fitted to justify the differences. The results are *shown* in Table 3. The first two columns of Table 3 shows that, during the period of impact resistance, the related diversified agglomeration has a significant positive effect on urban economic resilience, whereas the positive effect of unrelated diversified agglomeration is insignificant. We compare the regression coefficients and find that the positive effect of related diversified agglomeration on urban economic resilience is significantly larger than that of unrelated diversified agglomeration. Simply attributing the positive effect of diversified industrial structure to risk diversification is inappropriate and the mechanism behind deserve further exploration. On the one hand, compared with an unrelated industry, a related industry can more efficiently improve production efficiency by promoting the diffusion of knowledge and technology. On the other hand, the diversified industrial structure maintains a moderate cognitive distance, which can effectively prevent professional cognitive lock-in and risk contagion chain. As a result, the industrial structure with diversified and specialized attributes jointly alleviates the impact on the urban economy. Moreover, Tables 2 and 3 show that urban innovation has a significant positive effect on urban economic resilience, which helps to explain the positive effect of related diversified agglomeration. That is, compared with unrelated diversified agglomeration, related diversified agglomeration is more beneficial for technological innovation.

**Table 3:** The effects of two types of industrial diversification on urban economic resilience.

	the period of resistance to shock		The period of recovery and adjustment	
	(1)	(2)	(3)	(4)
*RV*	1.905** (2.21)		-6.218*** (-3.30)	
*UV*		1.171 (1.27)		-5.836*** (-3.54)
*Gover*	3.958 (1.50)	4.461* (1.67)	-3.524 (-0.70)	-3.799 (-0.76)
*Eco*	0.516 (1.52)	0.516 (1.49)	0.105 (0.11)	-0.091 (-0.09)
*Fdi*	3.247 (0.46)	-0.983 (-0.14)	34.760** (2.49)	36.880*** (2.64)
*Fix_cap*	-0.202 (-0.34)	-0.019 (-0.03)	-0.991 (-1.12)	-0.846 (-0.96)
*R&D*	0.165*** (2.84)	0.142** (2.46)	0.543* (1.92)	0.607** (2.14)
*Humcap*	-0.130 (-0.97)	-0.121 (-0.89)	0.103 (0.19)	-0.009 (-0.02)
*Rd*	0.188 (0.74)	0.165 (0.65)	0.022 (0.05)	-0.026 (-0.06)
*Popden*	0.357** (2.03)	0.489** (2.56)	-1.755 (-0.49)	0.154 (0.04)
*Market*	-0.248 (-1.33)	-0.275 (-1.42)	0.068 (0.10)	0.128 (0.19)
*_cons*	-5.933 (-1.37)	-5.932 (-1.32)	14.750 (0.66)	8.146 (0.37)
*N*	241	241	1205	1205
*R* ^*2*^	0.047	0.034	0.336	0.337

*Notes*: *t* statistics are shown in parentheses.

*, **, and ***indicate the significance of p-values at 10%, 5% and 1% level, respectively.

The last two columns of Table 3 show that, during the period of recovery and adjustment, related and unrelated diversified agglomerations have a significant negative effect on urban economic resilience. This result is consistent with the result of the overall diversified industrial agglomeration, indicating that both types of diversified industrial agglomeration depend on diversified production inputs, which is a huge burden on the urban economy during the period of recovery and adjustment. We compare the regression coefficients and find that the negative effect of related industrial diversified agglomeration is significantly greater than that of unrelated diversified agglomeration. One likely explanation is that, compared with the loosely connected unrelated diversified industrial structure, related diversified industrial structure with diversified and specialized attributes has higher requirements on production factors and thus put a bigger burden on the urban economy.

### 5.2 The effect of industrial agglomeration with different levels on urban economic resilience

Different types of industrial agglomeration can have different effects on urban economic resilience. An intuitive conjecture would be that different levels of industrial agglomeration can lead to different effects on urban economic resilience. To justify this conjecture, we categorize specialization agglomeration into a high level (denoted as *SP*_*H*_) and a low level (denoted as *SP*_*L*_) with the average industrial specialization agglomeration level in each year serving as the threshold [50]. Analogously, industrial diversified agglomeration is also categorized into a high level (denoted as *DIV*_*H*_) and a low level (denoted as *DIV*_*L*_). Meanwhile, we perform shrinkage treatment. The sample data are winsorized at 1% from both ends to eliminate the influence of extreme values on estimation. The regression results are shown in Table 4.

**Table 4:** The effects of industrial agglomeration with different levels on urban economic resilience.

	The period of impact resistance				The period of recovery and adjustment			
	(1)	(2)	(3)	(4)	(5)	(6)	(7)	(8)
*SP*_*H*_	-0.144 (-1.47)				0.051 (0.72)			
*SP*_*L*_		-0.206 (-0.88)				0.469* (1.86)		
*DIV*_*H*_			2.836* (1.69)				0.825 (0.46)	
*DIV*_*L*_				0.336 (0.35)				-7.768*** (-4.64)
*Gover*	-3.588 (-0.30)	5.565* (1.96)	7.594** (2.23)	0.074 (0.01)	3.479 (0.23)	-4.032 (-0.83)	-8.409 (-1.58)	-3.273 (-0.35)
*Eco*	0.811 (0.68)	0.391 (0.96)	1.471*** (3.20)	-1.078* (-1.86)	1.207 (0.45)	0.474 (0.41)	-1.591 (-1.41)	0.868 (0.49)
*Fdi*	23.830 (1.06)	-3.569 (-0.47)	2.257 (0.22)	1.567 (0.16)	59.490* (1.91)	25.680 (1.57)	59.010*** (3.34)	16.930 (0.83)
*Fix_cap*	-1.004 (-0.51)	-0.187 (-0.29)	-0.810 (-1.07)	0.880 (0.81)	0.378 (0.20)	-0.434 (-0.40)	-0.730 (-0.79)	-1.426 (-0.70)
*R&D*	0.094 (0.57)	0.151** (2.35)	0.071 (0.73)	0.259*** (3.60)	-0.629 (-1.05)	0.984*** (2.87)	0.350 (1.01)	1.242** (2.43)
*Humcap*	-0.327 (-0.98)	-0.080 (-0.52)	-0.313* (-1.77)	0.213 (0.92)	-0.882 (-1.20)	0.650 (0.85)	-0.053 (-0.10)	-0.420 (-0.33)
*Rd*	-0.015 (-0.02)	0.249 (0.91)	0.225 (0.66)	0.303 (0.71)	-0.467 (-0.55)	0.131 (0.23)	0.247 (0.50)	-0.309 (-0.30)
*Popden*	0.343 (0.70)	0.250 (1.13)	0.735*** (3.06)	0.304 (0.90)	2.553 (0.30)	-1.120 (-0.27)	4.322 (0.94)	-7.284 (-1.16)
*Market*	-1.003 (-1.27)	-0.190 (-0.89)	-0.594** (-2.20)	0.155 (0.54)	-2.535 (-0.70)	0.263 (0.36)	-0.735 (-0.87)	1.682 (1.53)
*_cons*	7.357 (0.35)	-3.125 (-0.67)	-16.520** (-2.39)	3.621 (0.48)	11.400 (0.16)	-3.093 (-0.12)	8.480 (0.29)	26.066 (0.70)
*N*	44	197	150	91	220	985	750	455
*R* ^*2*^	0.001	0.020	0.059	0.115	0.245	0.346	0.399	0.329

*Notes*: *t* statistics are shown in parentheses.

*, **, and ***indicate the significance of p-values at 10%, 5% and 1% level, respectively.

Table 4 shows that, during the period of impact resistance, the positive effect is significant for high-level diversified agglomeration but not for low-level diversified agglomeration, with the regression coefficient of the former being much larger than that of the latter. Therefore, high-level diversified agglomeration can have a much stronger effect on the urban economy regarding impact resistance. The difference can be attributed to the significant differences in the industrial scale, industrial productivity, and innovation ability between the two. Regardless of the level of specialization agglomeration, negative effects on urban economic to impact resistance are found. We compare the regression coefficients and find that the negative effect of high-level specialized agglomeration is weaker than that of low-level specialized agglomeration. In general, high-level industrial agglomeration can contribute more to urban economic resilience compared with low-level industrial agglomeration. During the recovery and adjustment period, the low-level diversified agglomeration has a significant negative effect, whereas high-level diversified agglomeration has an insignificant positive effect, with the regression coefficient of the former being much larger than that of the latter. The level of industrial agglomeration should be considered when the effect of diversified agglomeration on urban economic resilience is analyzed. Diversified agglomeration can be taken as a diversified investment. Low-level diversified agglomeration is in the accumulation stage when inputs outweigh outputs dramatically. In contrast, high-level diversified agglomeration can facilitate industrial reproduction through the antedate accumulation of production factors and thus help the urban economy to recover and adjust. High-level and low-level specialized agglomerations have positive effects on the recovery and adjustment of the urban economy, with a significant difference in effects. The reason is that, with the increase in the level of specialization agglomeration, the marginal effect of scale economy becomes less. The urban economy with low-level industrial specialization agglomeration can take the advantage of chain reaction to improve the scale economy in a short time to recover production effectively. In contrast, high-level industrial specialized agglomeration only has restrained resources to improve production efficiency. Thus, the effect on the urban economy is relatively limited.

Given that the industrial *agglomeration* level is important in analyzing the effect of diversified agglomeration on urban economic resilience, this study further categorizes the diversified industrial agglomeration into high-level related (denoted as *RV*_*H*_), high-level unrelated (denoted as *UV*_*H*_), low-level related (denoted as *RV*_*L*_), and low-level unrelated (denoted as *UV*_*L*_) according to the yearly thresholds of related and unrelated diversification agglomerations. The regression results are shown in Table 5.

**Table 5:** The effects of related and unrelated diversified agglomerations with different levels on urban economic resilience.

	the period of resistance to shock				The period of recovery and adjustment			
	(1)	(2)	(3)	(4)	(5)	(6)	(7)	(8)
*RV*_*H*_	1.980 (0.10)				-1.521 (-0.56)			
*RV*_*L*_		-9.934 (-0.36)				-10.378*** (-3.47)		
*UV*_*H*_			3.529 (1.52)				4.595* (1.84)	
*UV*_*L*_				0.551 (0.34)				-12.212*** (-5.59)
*Gover*	10.970*** (2.86)	-4.323 (-0.95)	7.140* (1.74)	1.378 (0.34)	-11.850** (-2.31)	4.296 (0.49)	-1.263 (-0.17)	-5.083 (-0.85)
*Eco*	1.116** (2.28)	-0.561 (-1.03)	1.227** (2.06)	-0.0008 (-0.00)	-1.325 (-1.08)	-0.193 (-0.12)	-2.419* (-1.79)	2.566* (1.87)
*Fdi*	8.233 (0.81)	-7.605 (-0.77)	-15.310 (-1.27)	3.667 (0.42)	39.200** (2.14)	22.920 (1.14)	57.33*** (3.31)	19.270 (0.86)
*Fix_cap*	-1.214 (-1.60)	0.928 (0.92)	-0.113 (-0.12)	0.500 (0.56)	-0.718 (-0.79)	-1.186 (-0.61)	-1.181 (-1.02)	0.258 (0.19)
*R&D*	0.155* (1.93)	0.123 (1.49)	0.117 (1.39)	0.169* (1.90)	0.789** (2.31)	0.574 (1.11)	0.492 (1.36)	0.767* (1.74)
*Humcap*	-0.314* (-1.82)	0.249 (1.15)	-0.104 (-0.48)	-0.077 (-0.42)	0.106 (0.20)	-0.370 (-0.31)	-0.324 (-0.48)	1.170 (1.27)
*Rd*	0.243 (0.77)	0.205 (0.44)	-0.147 (-0.34)	0.399 (1.11)	0.0579 (0.12)	0.310 (0.31)	0.383 (0.63)	-0.422 (-0.58)
*Popden*	0.177 (0.70)	0.678** (2.59)	0.958*** (3.27)	0.163 (0.53)	2.026 (0.49)	-9.099 (-1.44)	3.812 (0.82)	-4.058 (-0.77)
*Market*	-0.092 (-0.34)	-0.247 (-0.91)	-0.616* (-1.94)	-0.138 (-0.49)	-0.555 (-0.64)	0.821 (0.76)	0.071 (0.08)	0.707 (0.66)
*_cons*	-13.120 (-0.62)	14.310 (0.48)	-13.230* (-1.83)	-0.748 (-0.11)	17.610 (0.65)	43.676 1.13)	4.458 (0.15)	-1.328 (-0.04)
*N*	149	92	119	122	745	460	595	610
*R* ^*2*^	0.057	0.110	0.048	0.014	0.401	0.308	0.409	0.340

*Notes*: *t* statistics are shown in parentheses.

*, **, and ***indicate the significance of p-values at 10%, 5% and 1% level, respectively.

Table 5 shows that, except for low-level related diversified agglomeration, the other three categories of diversified agglomerations all have positive effects on urban economic resilience during the period of impact resistance. Compared with unrelated industrial diversification, related industrial diversification is at a lower level of risk diversification because of the correlated industrial structure and requires more production factors from the urban economy to achieve the dual characteristics of diversification and specialization. Compared with high-level related industrial diversification, low-level related industrial diversification has not yet formed a comprehensive industrial structure and thus requires more input for development. Therefore, when the urban economy experiences impact, low-level industrial diversification can hardly provide any support for impact resistance, and the production input required becomes the burden that can worsen the situation. Moreover, regardless of the type of diversified agglomeration, the effect of high-level agglomeration on urban economy resilience is always greater than that of low-level agglomeration, indicating that the established risk-diversification industrial structure can effectively contribute to impact resistance.

During the recovery and adjustment period, except for high-level unrelated diversified agglomeration, the other three categories of diversified agglomerations all have negative effects on urban economic resilience. The negative effects of low-level diversified agglomerations are significant. As discussed earlier, the *development* of diversified agglomeration relies on diversified production inputs. The low-level diversified industrial structure requires large production factors, which become burdens for the recovery and adjustment of the urban economy. In contrast, high-level agglomeration can make use of established industrial structure and antedate accumulation of technology as the basis of urban economic recovery and adjustment. The effect of high-level unrelated diversified agglomeration on urban economic resilience is significantly greater than that of high-level related diversified agglomeration. Similar to high-level specialization agglomeration, the high-level diversified agglomeration of related industries is also restrained by the diminishing marginal effect of the scale economy. The functioning of a high-level related industrial structure can be constrained by limited resources, whereas unrelated industrial diversification has lesser constraints and can allocate limited resources more efficiently to improve the urban economic situation.

### 5.3 The effects of dual industry agglomeration on urban economic resilience

The analysis above shows that different types and levels of industrial agglomeration have different effects on urban economic *resilience*. Thus, examining the combined effect of dual industrial agglomerations is intuitive. For example, does high-level dual industry agglomeration (the combination of high-level specialized agglomeration and high-level diversified agglomeration) have more enhancing effect on urban economic resilience? Therefore, we further categorize industrial agglomeration by types and levels simultaneously, leading to high specialization and high diversification (referred to as “high-high”), high specialization and low diversification (referred to as “high-low”), low specialization and high diversification (referred to as “low-high”), and low specialization and low diversification (referred to as “low-low”). For example Erdos, with the strategic shift of China’s energy resources and the development of the western region, the Erdos government vigorously develops industry, actively develops resource transformation industries, and strives to build a national-level energy and heavy chemical industry base. At the same time, it has promoted the concentration of industry to parks and industry to bases, building a circular industrial chain such as coal chemical industry, natural gas chemical industry, and chlor-alkali chemical industry. In addition, on the basis of maintaining the basic stability of the proportion of the secondary industry, Erdos has coordinated resources to accelerate the development of modern service industries, cultural tourism, finance, logistics and other industries have developed rapidly. In 2015, the tertiary industry accounted for about 40% of the regional GDP. Relying on its abundant energy reserves, Erdos first used specialized agglomeration to develop its industry, and then vigorously developed the tertiary industry on this basis, thus forming an industrial structure that is both highly specialized and diversified. The regression results are shown in Table 6.

**Table 6:** The effects of dual industry agglomeration on urban economic resilience.

	The period of impact resistance								The period of recovery and adjustment							
	(1)	(2)	(3)	(4)	(5)	(6)	(7)	(8)	(9)	(10)	(11)	(12)	(13)	(14)	(15)	(16)
*SP*_*HH*_	-0.412** (-2.49)								0.033 (0.32)							
*DIV*_*HH*_		6.223 (1.42)								9.509** (2.33)						
*SP*_*HL*_			0.180 (1.27)								0.117 (0.76)					
*DIV*_*HL*_				1.950 (0.45)								5.969 (1.06)				
*SP*_*LH*_					0.160 (0.48)								-0.199 (-0.79)			
*DIV*_*LH*_						1.802 (0.93)								-1.916 (-0.95)		
*SP*_*LL*_							-0.802* (-1.94)								1.486*** (2.82)	
*DIV*_*LL*_								-0.014 (-0.01)								-9.679*** (-5.03)
*Control variables*	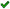	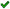	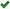	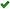	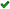	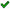	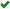	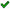	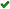	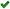	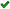	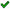	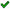	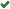	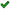	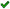
*_cons*	7.356 (0.30)	-12.960 (-0.47)	35.950 (1.15)	58.410 (1.84)	-9.448 (-1.47)	-13.350* (-1.72)	2.395 (0.33)	3.666 (0.44)	-68.170 (-0.67)	-114.700 (-1.13)	86.440 (0.55)	17.880 (0.12)	-12.590 (-0.40)	-8.686 (-0.28)	-2.690 (-0.05)	1.303 (0.02)
*N*	27	27	17	17	123	123	74	74	135	135	85	85	615	615	370	370
*R* ^*2*^	0.273	0.105	0.371	0.229	0.012	0.007	0.134	0.082	0.260	0.299	0.302	0.309	0.463	0.464	0.324	0.363

Table 6 shows that, during the period of impact resistance, the effect of diversified agglomeration on urban economic resilience is always superior to that of industrial specialized agglomeration, with higher levels of *agglomeration* showing more advantages. For the “low-low” category, specialized and diversified agglomerations do not contribute to impact resistance. Low-level specialized agglomeration cannot provide sufficient scale economy, and the chain industrial structure can lead to rapid risk contagion. Relying on the supply of urban economy, low-level diversified agglomeration cannot enhance urban economies that are resistant to impact. For the “high-high” category, specialized agglomeration shows a significant negative effect on urban economic resilience. The spiral risk contagion amplification mechanism of highly specialized industrial structure dominates the diminishing marginal effect brought by the gradually saturated scale economy.

During the period of recovery and adjustment, the effect of specialized and diversified agglomerations on urban economic resilience depends on the dual industrial agglomeration type of urban economy. For the “high-high” *category*, although both types of industrial agglomeration can bring positive effects for urban economic recovery, the effect of diversified agglomeration is much more significant. The reason is that, although high-level specialized and diversified agglomerations can provide the basis for the adjustment and recovery of the urban economy through antedated accumulated production factors, the marginal benefits of highly specialized industrial structures through the scale economy cannot outweigh the dynamic resource allocation of highly diversified industries structure through a multi-industrial network. For the “low-low” category, low-level diversified agglomeration undoubtedly has an adverse effect on the adjustment and recovery of the urban economy due to high demand for production factors. In contrast, low-level specialized agglomeration can use limited resources to form scale economy rapidly to contribute to the recovery of the urban economy.

The coordination effect between specialized and diversified agglomerations on urban economic resilience can also be found, particularly evident in the “high-low” and “low-high” categories. For unequal industrial *agglomeration*, the effect of low-level industrial agglomeration on urban economic resilience is always better than that of high-level industrial agglomeration during the period of impact resistance, except for the “low-high” category. The high-level industrial agglomeration can provide necessary production factors for the low-level industrial agglomeration to support its functioning in the adjustment and recovery of the urban economy. For the “low-high” category, low-level specialized agglomeration and high-level diversified agglomeration can bring positive effects to urban economic resilience during the period of impact resistance. The identification of the coordination effect justifies the necessity of analyzing dual industry agglomeration.

Similar to the analysis above, we further analyze the effect of related and unrelated diversified agglomerations on urban economic resilience by categorizing them into “high-high,” “high-low,” “low-high,” and “low-low.” The regression results are shown in Table 7. During the period of impact resistance, for the “high-high” and “low-high” categories, related and unrelated diversified agglomeration have positive effects on urban economic resilience. In contrast, for the “low-low” and “high-low” categories, unrelated diversified agglomerations show insignificant negative effects on urban economic resilience. Therefore, during the period of impact resistance, the effect of diversified agglomeration on urban economic resilience mainly depends on the level of unrelated diversified agglomeration. That is, if the risk diversification level of the urban economy is high, both types of diversified agglomerations can enhance the urban economy’s resistance to impacts. If the level of urban decentralization is low, both types of diversified agglomerations can produce negative effects.

**Table 7:** The effects of dual industry diversified agglomeration on urban economic resilience.

	The period of impact resistance								The period of recovery and adjustment							
	(1)	(2)	(3)	(4)	(5)	(6)	(7)	(8)	(9)	(10)	(11)	(12)	(13)	(14)	(15)	(16)
*RV*_*HH*_	0.922 (0.24)								-6.613* (-1.94)							
*UV*_*HH*_		4.693 (1.40)								-4.512 (-1.36)						
*RV*_*HL*_			9.344** (2.43)								5.180 (1.19)					
*UV*_*HL*_				-0.048 (-0.02)								-10.220** (-2.38)				
*RV*_*LH*_					0.227 (0.08)								-9.342* (-1.72)			
*UV*_*LH*_						4.019 (1.24)								16.620*** (3.72)		
*RV*_*LL*_							1.283 (0.61)								-12.220*** (-3.02)	
*UV*_*LL*_								-0.979 (-0.47)								-15.640*** (-5.21)
*Control variables*	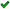	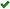	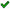	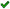	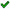	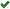	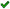	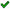	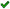	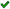	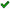	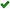	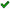	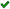	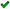	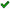
*_cons*	-27.820** (-2.41)	-28.490*** (-2.84)	-18.020 (-1.37)	-1.340 (-0.11)	4.897 (0.58)	-1.249 (-0.13)	-6.312 (-0.59)	-3.802 (-0.37)	2.113 (0.06)	-13.040 (-0.39)	23.370 (0.47)	57.640 (1.16)	130.400 (1.08)	57.670 (0.50)	-9.243 (-0.18)	-35.800 (-0.72)
*N*	82	82	67	67	37	37	55	55	410	410	335	335	185	185	275	275
*R* ^*2*^	0.093	0.117	0.086	0.011	0.227	0.269	0.059	0.063	0.485	0.482	0.342	0.353	0.321	0.371	0.332	0.384

The mechanisms of related and unrelated diversified agglomerations on urban economic resilience become more complicated during the period of recovery and adjustment. For the two equal dual agglomerations, “high-high” and “low-low,” the diversified agglomerations of related and unrelated industries have negative effects on urban economic resilience. For the “low-low” category, production factors squeezed by the diversified agglomeration can be unbearable for the urban economy during the period of recovery and adjustment. For the two non-equal dual agglomerations, “high-low” and “low-high,” high-level diversified agglomeration is always better than low-level diversified agglomeration on urban economic resilience, which is the opposite to the development model of “specialization-diversification” dual industry agglomeration. One likely explanation is that low-level diversified agglomeration is still in the period of squeezing production factors, which will worsen the constraint on resources for the urban economy. However, high-level diversified agglomeration can effectively allocate resources through a systematic industrial network and utilize accumulated production factors to support urban economy’s recovery and adjustment.

## 6 Conclusions

In this study, we use the sensitivity index method to measure the economic resilience of 241 cities at the prefecture-level and above in China and comprehensively analyze the effects of industrial specialized and diversified agglomerations, as well as two sub-type diversified agglomerations, on urban economic resilience. We scrutinize the heterogeneity in effects under the conditions of different single and dual agglomeration levels. The main conclusions are as follows: (1) During the period of impact resistance, diversified agglomeration, especially related diversified agglomeration, can enhance urban economy’s resistance to impacts, whereas specialized agglomeration cannot contribute to impact resistance. During the period of recovery and adjustment, specialized agglomeration becomes more beneficial to urban economy resilience, whereas diversified agglomeration, especially related diversified agglomeration, becomes unbeneficial. (2) From the perspective of the difference in agglomeration levels, during the period of impact resistance, high-level industrial agglomerations are generally more contributive to urban economy resilience compared with low-level industrial agglomerations. The positive effect of high-level diversified agglomeration is significant, whereas the positive effect of low-level diversified agglomeration is insignificant. Regardless of the level of specialized agglomeration, it has an adverse effect on the urban economy’s impact resistance. During the period of recovery and adjustment, the negative effect of low-level diversified agglomeration is significant, whereas the positive effect of high-level diversified agglomeration becomes insignificant. Specialized agglomerations have positive effects on the adjustment and recovery of the urban economy regardless of the agglomeration level, with a significant difference in effects. (3) From the perspective of difference in agglomeration levels of dual agglomerations, during the period of impact resistance, the effect of diversified agglomeration on urban economic resilience is always superior to that of specialized agglomeration regardless of the types of dual agglomerations, with higher agglomeration levels showing more advantages. For the “low-low” dual industry agglomeration, neither specialized agglomeration nor diversified agglomeration can be contributive to impact resistance. For the “high-high” dual industry agglomeration, specialized agglomeration has a significant negative effect on urban economic resilience. During the adjustment and recovery period, for the two balanced dual industrial agglomerations, “high-high” and “low-low,” related and unrelated diversified agglomerations have negative effects on urban economic resilience. For the two unbalanced dual industrial agglomerations, “high-low” and “low-high,” high-level diversified agglomerations always contribute more to urban economic resilience than low-level diversified agglomerations.

With increasing uncertainty and instability worldwide, the development of emerging markets encounters many problems and challenges. Enhancing urban economic resilience (i.e., resistance to impacts and adjustment and recovery after impacts) is of vital importance to developing countries, such as China. The empirical results of the effects of industrial agglomerations on urban economic resilience lead to a few policy implications as follows. First, the government can promote the development of industrial agglomeration to enhance urban economic resilience. Being aware that different types of industrial agglomerations have different effects depending on the stage of the crisis is important. Therefore, policy-making should be in accordance with the specific situation of the urban economy. Meanwhile, the coordination between different types of industrial agglomerations should be considered. Second, different levels of industrial agglomerations have different effects on urban economic resilience at different stages of a crisis. Therefore, the government should consider the current industrial agglomeration levels when making policies, especially when the urban economy undergoes external impacts. Third, in the development of industrial agglomeration, the urban economy should seek a good balance between specialized and diversified agglomerations. Cities with high-level specialized agglomeration can introduce “new economy” sectors, develop high-tech industries, increase the level of diversified agglomeration, and strengthen the spillover effects of Jacobs externality, thereby promoting industry diversification and advanced development of the structure. Cities with high-level diversified agglomeration can build high-tech parks, create a highly specialized regional environment, and strengthen the localized economy generated by specialized agglomeration.

## Supporting information

S1 Date (XLSX)
